# Attention-Dependent Physiological Correlates in Sleep-Deprived Young Healthy Humans

**DOI:** 10.3390/bs11020022

**Published:** 2021-02-05

**Authors:** Valentina Cesari, Elena Marinari, Marco Laurino, Angelo Gemignani, Danilo Menicucci

**Affiliations:** 1 Department of Surgical, Medical and Molecular Pathology and Critical Care Medicine, University of Pisa, 56126 Pisa, Italy; valentinacesari91@gmail.com (V.C.); elenamarinari2@gmail.com (E.M.); angelo.gemignani@unipi.it (A.G.); 2 Institute of Clinical Physiology, National Research Council, 56124 Pisa, Italy; laurino@ifc.cnr.it

**Keywords:** cognitive functions, attention, physiological signals, workload, sleep deprivation

## Abstract

Cognitive functions could be specifically altered but masked from the unspecific effect of workload, a common factor affecting cognitive functions that modulate peripheral outputs. To identify workload-related and specific, task-dependent components, physiological correlates of cognitive functioning were derived by studying 15 healthy volunteers performing attentional tasks in baseline and post-sleep-deprivation conditions (one week interval). Sleep deprivation was introduced to increase workload. We performed recordings of heart pulse, facial temperature, and head movements during tasks assessing attentional network efficiency (ANT, Attentional Network Task; CCT, Continuous Compensatory Tracker) workload assessments after execution of tasks. Changes in cognitive and physiological indices were studied in both conditions; physiological correlates of cognitive performance were identified by correlating changes from baseline to post-sleep-deprivation condition of task indices with those of physiological measures after correction for between-conditions workload changes. We found that mental and physical demands of workload increased after sleep deprivation. We identified no changes in cognitive and physiological indices across conditions; specific physiological correlates of attentional systems, as indicated by the negative correlation between changes in ANT-alerting and changes in amplitude of head movements and the positive correlation between changes in CCT-speed indexing alertness and changes in facial temperature.

## 1. Introduction

The daily living of human beings is driven by cognitive processes related to the transformation, reduction, elaboration, storage, and recovery of sensory input in the real world [1]. The functioning of distinct cognitive functions is based on specific brain networks and gives rise to distinguishable activations; however, a common factor affecting cognitive functions is workload, the multidimensional construct quantifying the level of mental and physical effort put forth by a performer in response to cognitive tasks. The evaluation of workload spans from classical neurocognitive tests to dynamic situations such as aviation and driving [2]; however, it is usually considered a property of an individual’s attitude toward a demanding situation rather than to a task [3,4].

Workload is sustained by arousal and it is described as an indicator of pressure on working memory [5]. The arousal during workload implies an autonomic activation involved in non-consciously coordinated bodily responses for homeostasis [6]. Autonomic arousal is sustained by the activity of the central autonomic network, that controls electrophysiological changes related to cognitive and emotional processing [7]. Besides workload-related effects, several studies have highlighted specific neurofunctional patterns associated with cognitive tasks involving different domains. Concerning the three networks of attention, Posner and Petersen [8] identified the alerting, orienting, and executive networks. Both variations in arousal and in individual cognitive functions give rise to changes in peripheral autonomic outputs [9] that we investigate in the present work.

From a methodological perspective, cognitive functioning has been studied by taking advantage of intra-subject variability, which has been widely assessed in the context of sleep deprivation, that acts as a reliable paradigm to induce acute stress response. Indeed, acute and chronic sleep deprivation represents a tangible risk that exposes subjects to stressful conditions in modern society, posing high and significant risks for quality of life and psycho-physical wellbeing, including cognitive performance degradation [10]. Both acute total sleep deprivation and chronic sleep restriction increase homeostatic sleep—process S—leading to sleep debt. Process S augments during wakefulness and decreases during sleep time [11]; this increasingly impairs cognitive functions such as attention, cognitive speed, and memory during wakefulness [12]. For this reason, several studies used an acute sleep deprivation model to understand its impact on various cognitive domains and on subjective workload. Indeed, acute sleep deprivation might negatively influence some aspects of cognitive functioning, in particular vigilance, which, if lowered, increases the risk of accidents [13]. It has been reported that subjects perceived high workload after a night of sleep loss, and that factors external to tasks concur to increase perceived workload [14,15].

In these studies, the autonomic outputs are often assessed, since higher mental workload is associated with a decrease of parasympathetic (“rest or digest”) autonomous nervous system activity and an increase in sympathetic (“fight or flight”) activity [16].

These changes in autonomous nervous system activity have been estimated with several peripheral physiological measures such as heart rate, skin conductance, and peripheral temperature [17] during tasks assessing different cognitive domains. For example, there is a positive association between cognitive load, level of glucose and oxygen in the brain [18], and forehead temperature [19,20]. An increase in heart rate with increasing difficulty in decision making and level of attention [21,22] and a decrease in heart rate variability with increasing difficulty in response inhibition and memory [23,24] have also been documented. Regarding skin conductance, its increase has been detected during the performance on attention, memory, vigilance, and visual tracking tasks [25,26,27].

Moreover, different sensors placed in different putative sites have been reported to be used [19,28,29]. Herein, we assemble all sensors in the periocular area—skin temperature at cheekbones and forehead [19], heart pulse at the glabella (the small area between the eyebrows and above the nose) [30], and head movements from a sensor integral with the head [31].

In summary, many studies have used specific cognitive tasks to establish cognitive-related physiological outcomes. Several studies, taking into account subjective workload, have shown that physiological measures depend on the degree of subjective perceived difficulty while performing tasks. In fact, subjective workload measurement has been reported to be often dissociated from cognitive performance (e.g., subjects report high cognitive demand but cognitive performance is not negatively altered [32]). This evidence implies the urge to identify the modulation of specific cognitive functions on the peripheral physiological signals, as suggested by the specific central networks sustaining the different cognitive domains. In fact, the identification of specific cognitive correlates is essential for the detection and the rehabilitation of those functions that could be specifically altered but masked from the unspecific effect of workload. To this aim, we studied the peripheral physiological correlates (heart rate, head movements, and facial skin temperature) from sensors placed in the periocular area in subjects undergoing different cognitive tasks involving attentional systems at baseline and post-sleep-deprivation. We assessed the perceived workload in performing the tasks in these two different conditions and we studied associations between changes from baseline to sleep deprivation of physiological measures and cognitive indices correcting for the workload changes. We chose to administer the Attentional Network Task (ANT, [8,33]) and Continuous Compensatory Tracker (CCT, [34,35]) tests to better characterize the independence of different systems functioning (alerting, orienting, executive networks), that proved to be differently affected by sleep deprivation [36]. To assess subjective workload after cognitive tests, we used the NASA Task Load index (NASA TLX) scale [37], since previous studies reported it to be to a sensible scale to detect changes in the degree of experienced workload [14,38]. The use of sleep deprivation allowed us to induce a transient and reversible cognitive alteration that can be studied to compare specific attentional alterations.

While performing different tasks in the same subjects, we had the opportunity to separate the common peripheral response over the tasks, putatively related to cognitive workload, from that one characterizing specific cognitive functions engaged in each specific task. The recognition of peripheral response changes, as indices of cognitive alterations, could be used to detect attentional decrements during everyday life activities, such as driving, without deviating subjects to a cognitive task administration.

## 2. Materials and Methods

### 2.1. Participants

Fifteen healthy young volunteers (nine females and six males; mean age ± SD: 24.5 ± 2 years) were enrolled for this experimental protocol. Subjects eligible for inclusion met the following criteria: absence of psychiatric symptoms, as assessed by Symptom Checklist-90-Revised [39,40]; absence of sleep-wakefulness disorders, as assessed by Insomnia Severity Index (ISI) [41,42] and Epworth Sleepiness Scale (ESS) [43,44]; absence of organic pathologies and psychotropic addiction, as assessed by a qualitative anamnestic questionnaire; normal or corrected vision; age between 18 and 35 years old.

### 2.2. Experimental Protocol

**Figure 1:**
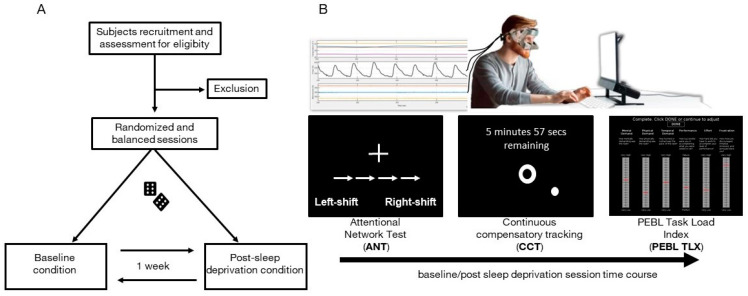
Experimental protocol. (**A**). Flow chart of experimental protocol. (**B**). Above, a sketch of the online recording of facial temperature, head movements, and heart pulse by means of the PERFORM system while the subject is sitting in front of a PC; below, the schematic representation of the experimental timeline for both baseline and post sleep deprivation conditions.

The experimental protocol consisted of two sessions (Figure 1A), which were randomized and balanced across participants and which took place with a one-week interval, at least. Each session started at 6 pm and each volunteer was tested individually. The laboratory room was temperature-controlled (22 °C).

Two cognitive tests followed by a perceived workload assessment were completed both in the baseline session and the post-sleep-deprivation session; Figure 1B. Sleep deprivation lasted from 8:00am of the day before the session to beyond the end of the session (about 8:00 pm)—a total of 36 hours. To ensure that volunteers had normal sleep before the baseline session, sleep monitoring was accomplished by actigraphy registration and by filling out a sleep diary for two days before each session [45,46]. Actigraphic monitoring was also used to ensure volunteers were completely sleep-deprived in post-sleep-deprivation sessions; any sleep episode implied the exclusion from the study. For this double purpose, participants wore an ActiGraph wGT3X-BT (ActiGraph, Pensacola, FL, USA) placed on their non-dominant wrist. Data were analyzed and visually inspected using Actilife software (version 6.11.9).

For each session, volunteers underwent the Attentional Network Task (ANT) and Continuous Compensatory Tracker (CTT) task implemented in the Psychology Experiment Building Language (PEBL) software and administered on a PC monitor (60 cm was the distance from the 27-inch screen to the eyes, screen resolution 1024 × 768). The time for performing ANT and CTT tasks was 15 and 10 min, respectively. We administered each task consecutively, without any intervals. To this purpose, we chained the task using the setting options given by PEBL software. In the PEBL software, subjects are assumed to perform a standardized training session to familiarize with task instructions and to prevent bias effects attributable to the order of the sessions. During the cognitive assessment, participants had to wear the Psychophysiological sEnsoRs Mask FOr Real-Life Cognitive Monitoring (PERFORM), a periocular sensorized mask for biosignal acquisition [29]. Thus, the perceived workload was assessed immediately after the completion of the PEBL cognitive tasks for both conditions by using PEBL NASA Task Load Index (PEBL TLX) scale [47]. The overall duration of the session was about 30 min.

### 2.3. Cognitive Assessment

#### 2.3.1. Psychology Experiment Building Language (PEBL) Attentional Network Task

The attentional network task (ANT) aims to assess the functioning of alerting, orienting, and executive control attentional networks [8,33]. Participants are supposed to determine the direction of the central arrow in a set of five, while ignoring the directions of the surrounding arrows. To indicate the correct direction of the central arrow, participants have to press the corresponding button on the keyboard. In the current study, the PEBL version of the Attentional Network Test was used and, according to Fan et al. [33], we considered the correct trials (i.e., not considering the incorrect answers) for the performance indices calculation, represented by (1) alerting index, (2) orienting index, and (3) conflict index.

The efficiency of the alerting network is examined by changes in reaction time (RT) resulting from a warning signal. The alerting index is calculated by subtracting the mean RT of the double-cue condition from the mean RT of the no-cue condition [36].

The efficiency of orienting is examined by changes in the RT that accompany cues indicating the location in which the target will occur. The orienting index is expressed as the difference between the mean RT of the items in a central cue condition (“central cue”) and the average RT of the items in a spatial cue condition (“spatial cue”) [36].

The efficiency of the executive network is examined by requiring the participant to respond by pressing two keys indicating the direction (left or right) of a central arrow surrounded by congruent, incongruent, or neutral flankers. The conflict index is calculated by subtracting the mean RT of congruent flanking conditions from the mean RT of incongruent flanking conditions [36].

#### 2.3.2. PEBL Continuous Compensatory Tracker

The Continuous Compensatory Tracking (CCT) is a cognitive test originally developed to assess alertness and vigilance [34,35], and also used to assess sustained attention. Participants have to continuously adjust the position of a pointer to overlap it to a target during eight consecutive trials (from T1 to T8). The pointer is under randomly directed forces that need to be continuously compensated [47]. In the current study, the PEBL version of the CCT task was used to assess vigilance by means of two indices—CCT deviation and CCT speed.

The degree of adaptation was assessed by considering the changes of the deviation and speed from the beginning (T1) to the end (T8) of the task:

- CCT deviation. The median of spatial displacements between the target position and the pointer was calculated for each trial (lower values of median deviation correspond to higher accuracy of task performance) and CCT deviation was estimated as the displacements change from the first to the last trial.
- CCT speed. The mean of mouse velocity over the task was calculated for each trial and CCT speed was estimated as the speed change from the first to the last trial. Mouse velocity should indicate the degree of the subject’s reactivity toward the task; higher values correspond to a higher degree of reactivity for compensating random motion of the pointer.

#### 2.3.3. PEBL NASA Task Load Index (PEBL TLX)

The NASA Task Load Index [37] is a self-report multidimensional scale aiming at providing an overall perceived workload score, based on a weighted average of six subscales. The subscales are mental demand, physical demand, temporal demand, own performance, effort, and frustration. Subjects have to rate the perceived workload experienced during the previous completion of cognitive tasks by choosing a score ranging from 0 to 100 for the six subscales; higher values indicate greater perceived workload. In the present study, the perceived workload rating was assessed by using the version of NASA TLX on PEBL software (PEBL TLX) [47].

### 2.4. Physiological Assessment

During cognitive assessment, participants wore the Psychophysiological sEnsoRs Mask FOr Real-Life Cognitive Monitoring (PERFORM), a validated multi-sensorized wearable and non-obtrusive mask [29] able to detect, record, and analyze the following physiological signals from a set of dry electrodes placed over the periocular area:

- Facial temperature signals, recorded at 1Hz sampling rate from sensors placed over the left and right zygomatic muscle and the left and right forehead;
- Heart pulse, recorded at 100 Hz sampling rate with a photoplethysmograph sensor placed over glabella (the area between the eyebrows and above the nose);
- Head movements signal recorded at 100 Hz sampling rate from a 3-axial accelerometer placed over the left side of the mask.

For each signal, peripheral measures were extracted to study how performing a cognitive task could change the peripheral outputs. In accordance with the physiological nature of each measurement, we obtained a time series of measures from the beginning to the end of each cognitive task. Thus, as effective parameters associated with the performed task, we considered the changes of each extracted measure from the beginning of the task (average of the measure over the first tenth of its time series) to its end (average of the measure over the last tenth of its time series).

From the facial temperature time series we considered:

- MaxT, defined as the maximum of the four temperature changes calculated between the beginning and the end of the task;
- zfT, defined by comparing the aforementioned T changes at the forehead vs. those at the cheekbones (zfT = ΔT_z_ − ΔT_f_ where ΔT_z_ is the average changes over the two forehead sensors, and ΔT_f_ is the average changes over the two cheekbones sensors).

From the heart pulse time series, we obtained the pulse to pulse time interval series [48], which allowed us to estimate the changes of heart rate (HR), defined as the rate change calculated between the beginning and the end of the task.

From the head movements time series, we obtained an integrated measure of head movements from the variance of the combined three axial oscillations calculated within consecutive 1 s windows. We estimated the head movement amplitude (HMA) as the changes between the beginning and the end of the task of this measure.

### 2.5. Statistical Analysis

This work aimed to identifying specific physiological correlates of performing tasks mainly involving a unique attentional component. We used sleep deprivation to manipulate workload in order to remove the effect of general workload on physiological responses. We formulated the following hypothesis (Ha) to be tested against the null hypothesis (H_0)_:

- H_0_ = no significant correlations between cognitive and physiological indices changes from baseline to post-sleep-deprivation condition after correcting for workload. Correlation between variables is = 0;
- H*a* = significant correlations between cognitive and physiological indices changes from baseline to post-sleep-deprivation condition after correcting for workload. Correlation between variables is <0 or >0.

H_0_ is rejected if Sig < α, where< α = 0.05

Accordingly, we analyzed the data by following two main steps:

(1) identifying sleep deprivation effects on the perceived workload;
(2) identifying physiological correlates of intra-subject cognitive performance changes (from baseline to post-sleep-deprivation) in the different cognitive tasks after removing the contribution of workload changes.

For step (1), PEBL TLX subscales differences between sleep deprivation and baseline sessions were assessed with a two-tailed Wilcoxon signed-rank test. Non parametric tests were used since they allowed studying of parameters irrespective of normality. Indeed, variables in this study were heterogeneous (from psychometric score to physiological parameters); some were skewed and some varied over a span of a few discrete values. We estimated effect size for non-parametric tests using the following formula:

$$r=\frac{Z}{\sqrt{N}}$$

in which *N* is the total number of observations on which Z score is based [49].

For step (2), physiological correlates of intra-subject cognitive performance changes were identified by correlating the changes of cognitive task indices and physiological measures from baseline to post-sleep-deprivation. To remove the contribution of workload changes from correlation values between cognitive indices and physiological measures, the partial correlations (r_p_, partial ranks correlation) were calculated by controlling for those workload subscales that reached the statistical significance at step (1).

The Yekutieli and Benjamini procedure [50] for controlling the false discovery rate (FDR) of the family of hypothesis tests concerning all physiological features correlated with each cognitive task index was applied. The false discovery rate was set equal to 0.05 and adjusted *p*-values were calculated.

## 3. Results

### 3.1. Perceived Workload Changes from Baseline to Sleep Deprivation

The perceived workload measured after tasks was different between conditions for mental and physical demand subscales; mental and physical demands increased in the post-sleep-deprivation condition (*p* = 0.04 and *p* = 0.01, respectively). Table 1 provides statistics for each PEBL TLX subscale.

### 3.2. Physiological Correlates of Attentional Systems’ Functioning

Regarding attentional and physiological indices, no significant differences between conditions was identified (Supplementary Tables S1–S4).

**Figure 2:**
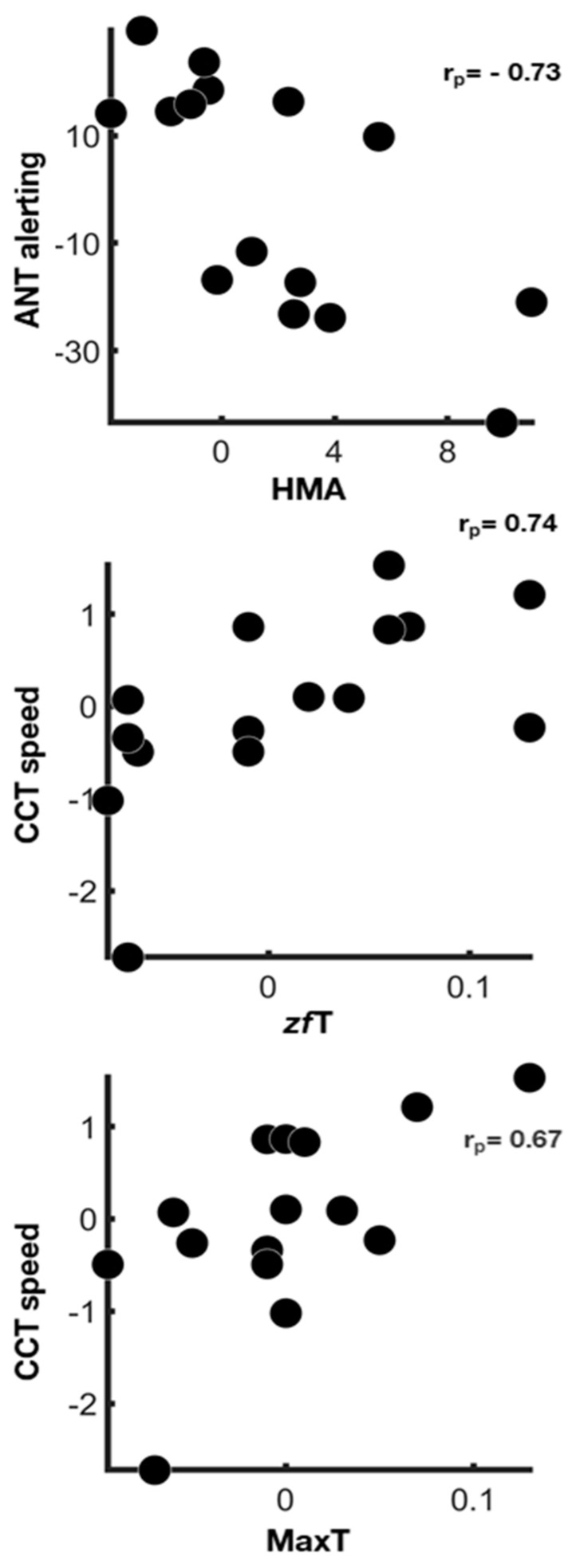
Physiological correlates of attentional systems functioning. Scatter plots show associations between physiological measures and cognitive indices changes from baseline to post-sleep-deprivation condition after the correction for TLX mental and physical demand (r_p_, partial rank correlation coefficient—false discovery rate < 0.05).

However, significant associations were found between cognitive indices and physiological measures after removing the putative linking effect related to the changes of perceived workload (Figure 2).

## 4. Discussion

Our study aimed to identifying peripheral physiological correlates of attention components in healthy subjects. A within-subject study using sleep deprivation as a stressor [12] was used to induce changes in individual cognitive performances. Each participant underwent two experimental sessions and one of which took place after one night of sleep deprivation. For each session, physiological signals were recorded using the PERFORM [29] during the completion of two cognitive tasks assessing attentional networks efficiency. After cognitive tests, an evaluation of the perceived workload was performed. The evaluation of perceived workload allowed us to get an estimate of its contribution to AT functioning changes from the baseline to the post-sleep-deprivation condition and to remove its unspecific contribution to the physiological/cognitive relationships.

Herein, physiological signals were derived from peri-ocular sites by using the PERFORM [29], a system conceived to be used in VR headset.

### 4.1. Sleep Deprivation Increases Mental and Physical Demands

Our results highlighted an increased mental and physical demand in the post-sleep-deprivation condition as compared to baseline. Several studies reported that the progress of prolonged wakefulness leads to a gradual increase in the homeostatic biological drive [51] that consequently augments the state of sleepiness. In this context, perceived workload increase after a period of prolonged wakefulness is reported [14,52] and explained as a counterbalancing response to a fatigued state. Liu and colleagues [38] reported a general increase in all workload subscales after 32 hours of sleep deprivation in a sample of expert airplane pilots after simulated flight tasks. Tomasko and colleagues [14] assessed workload in sleep-deprived medical students after laparoscopic-simulated tasks, while Fairclough and colleagues [53] did the same in sleep-deprived subjects after primary driving tasks and both highlighted increased scores for all NASA TLX subscales, except for the mental demand one.

Our findings partially agree with the previous studies, since perceived workload increase was limited to mental and physical demand subscales. It is worth noting that previous studies enrolled selected group of participants for their investigations, thus administering tasks intimately linked to the subjects’ activities of daily living (i.e., surgical task for surgeons, simulator flight task for expert flyers), whereas, in the present research, tasks were not part of everyday life and they could have represented a novel experience. The novelty of the tasks, the effort to comprehend instructions, and the lack of similarity with activities of daily living could represent additional mental stressors that are exacerbated by sleep deprivation. However, it is worth noting that the scores of cognitive tasks did not show significant differences between conditions (baseline and post-sleep deprivation). In this context, it is possible to suggest that the higher the perceived workload, the higher the effort to counteract fatigue to guarantee an adequate cognitive performance. Interestingly, cognitive task performance and physiological measures did not differ between baseline and post-sleep deprivation conditions, though participants reported higher perceived workload for mental and physical demand. Although these results could appear counterintuitive, previous studies reported dissociation between subjective workload and effective performance [14,32]. It is possible to suggest that subjective perception of high workload after an acute period of sleep loss could be a product of the complex mechanism to counteract sleep-related fatigue during prolonged wakefulness, which helps subjects perform tasks efficiently. However, it is important to point out the difference between acute and chronic sleep deprivation in the context of cognitive performance. Acute sleep deprivation might activate mechanisms to counteract detrimental effects ascribable to a few nights of sleep loss, in order to maintain adequate cognitive performance, while chronic sleep deprivation could permanently degrades cognitive abilities, especially the higher level ones [54].

### 4.2. The Physiological Correlates Differ between Attentional Systems’ Functioning

Our results highlighted the associations between changes from baseline to post-sleep deprivation conditions of physiological measures and attentional indices. These associations were putatively sustained by different factors—the general increase of workload and the modulation of the attentional systems specifically involved in each task. Most of the associations identified between physiological measures and attentional indices also held true after mental and physical demands correction, suggesting a strong contribution of specific attentional systems on physiological reactions.

As regards the alerting efficiency, a higher reactivity of this system in the post-sleep deprivation condition was associated with a decreased amplitude of head movements. Accordingly, a study of head movements during surgical tasks has shown that less head movement amplitude during a laparoscopic simulation was associated with better learning [55]. Our results sustain the hypothesis that fewer head movements are related to more precise and more focused performances during tasks.

Concerning vigilance during the visuo-motor tracking task, a higher reactivity (CCT speed) after sleep deprivation was shown in the participants who displayed greater temperature changes from the beginning to the end of the task between cheekbones and forehead sensors (zfT) and between all facial sensors. Classically, temperature fluctuations during tasks are associated with task performance. It has been reported that brain metabolism increase during cognitive tasks implies more heat production [20]. As some blood vessels connect facial tissues with the brain [56], metabolic brain changes cause variations in the peripheral skin temperature that we were able to detect.

## 5. Conclusions

This integrated evaluation of attentional systems using subjective, behavioral, and physiological measures allowed us to gain a better comprehension of attentional systems and their relative physiological changes after sleep deprivation. An augmented perceived physical and mental demand was shown in the post-sleep deprivation condition. Head movements and temperature variations were revealed to be sensitive to changes occurring during specific cognitive performance involving different attentional systems in sleep deprivation as compared to baseline.

This study has some limitations that should be addressed in future research. The small sample size and its homogeneity with respect to ethnicity and age could have precluded robust conclusions. Regarding statistical analysis, the choice of partial correlations to control the effect of workload was importantly limited by the low sample size. Moreover, correlation analysis did not indicate direction of interaction and could have been sustained by some latent factor modulating both measures. In this light much work would be needed for confirming and better specifying the associations suggested by our data. Also, the psychometric scales to ascertain the absence of sleep disorders did not cover all sleep disorders, such as restless leg syndrome; the Pittsburgh Sleep Quality Inventory should be used to prevent this issue of inclusion/exclusion. Finally, the sleep deprivation session was not conducted in a controlled-laboratory room.

## Figures and Tables

**Table 1:** PEBL Task Load Index.

PEBL NASA Task Load Index (PEBL TLX)	Condition	m ± s.e.	Δ (m ± s.e.)	Sum of Positive Ranks	*p*-Value	Effect Size
Mental demand	B	27 ± 4.82	11.66 ± 5	24	0.04 *	−0.37
	D	39 ± 5.42				
Physical demand	B	18 ± 2.71	21 ± 7	9	0.01 **	−0.46
	D	39 ± 6.62				
Temporal demand	B	43 ± 6	5.33 ± 8.41	46.5	0.70	−0.06
	D	48 ± 7				
Frustration	B	39 ± 4.29	3.66 ± 5.46	35.5	0.47	−0.12
	D	42 ± 4.36				
Effort	B	33 ± 5.36	14.66 ± 8	27.5	0.116	−0.28
	D	48 ± 7				
Performance	B	24 ± 6	5.66 ± 10	36	0.50	−0.12
	D	30 ± 7				
Overall workload	B	31 ± 2	10 ± 5	90.5	0.08	−0.31
	D	41 ± 4.35				

B, baseline condition; D, sleep-deprived condition; Δ = D − B; * Wilcoxon signed rank test with *p* < 0.05; ** Wilcoxon signed rank test with *p* < 0.01.

## Data Availability

Data is available on request to the corresponding author (D.M.).

## Supplementary Materials

The following are available online at https://www.mdpi.com/2076-328X/11/2/22/s1, Table S1. Attentional Network task. Table S2. Continuous Compensatory Tracker. Table S3. Physiological parameters recorded during the Attentional Network Task. Table S4. Physiological parameters recorded during the Continuous Compensatory Tracker.
