# Supplementary material for: Attention-Dependent Physiological Correlates in Sleep-Deprived Young Healthy Humans
Source: Behav Sci (Basel). 2021 Feb 5;11(2):22. doi: 10.3390/bs11020022 (PMC7915657; doi:10.3390/bs11020022)
Supplement: Supplementary file 1 [file behavsci-11-00022-s001.pdf]

DESCRIPTIVE AND INFERENTIAL STATISTICS FOR BETWEEN CONDITIONS INDICES

Table S1: Attentional Network task

Attentional Network Task indices	Condition	m ± s.e.	Δ (m ± s.e.)	Sum of positive ranks	p-value*	Effect size
**ANT Alerting**	**B**	24.4 ± 5.08	8.38 ± 6.82	81	.233	-0.21
	**D**	32.8 ± 5.89				
**ANT Orienting**	**B**	38.9 ± 5.43	4.2 ± 5.7	73	.460	-0.134
	**D**	43.16 ± 8.41				
**ANT Conflict**	**B**	90.5 ± 6.15	-5.4 ± 7.3	49	.532	-0.114
	**D**	85.09 ± 5.89				

B=baseline condition **D**=sleep deprived condition; **Δ=** D-B; *Wilcoxon signed rank test.

Table S2 - Continuous Compensatory Tracker

Continuous Compensatory Tracker (CCT) indices#	Condition	m ± s.e.	Δ (m ± s.e.)	Sum of positive ranks	p-value*	Effect size
**CTT Speed**	**B**	-0.05 ± 0.166	-0.02 ± 0.34	46	.426	-0.14
	**D**	-0.08 ± 0.33				
**CTT deviation**	**B**	-1.16 ± 0.67	-11.16 ± 11.52	67	.69	-0.07
	**D**	-12.33 ± 11.41				

[#] changes from the beginning (T1) to the end (T8) of the task; **B**=baseline condition. **D**=sleep deprived condition; **Δ=** D-B; * Wilcoxon signed rank test.

Table S3 - Physiological parameters recorded during the Attentional Network Task

Physiological parameters[#]	Condition	m ± s.e.	Δ (m ± s.e.)	Sum of positive ranks	p-value*	Effect size
zfT	B	0.061 ± 0.02	0.03 ± 0.03	68.5	.315	-0.18
	D	0.092 ± 0.035				
MaxT	B	0.26 ± 0.035	0.048 ± 0.040	59.5	.327	-0.17
	D	0.31 ± 0.03				
HR	B	-0.005 ± 0.13	-0.002 ± 0.013	58	.910	-0.02
	D	-0.007 ± 0.007				
HMA	B	0.12 ± 0.58	1.87 ± 1.11	81	.233	-0.21
	D	1.99 ± 0.88				

[#] for each measure, the changes from the beginning of the task (average of the measure over the first tenth of its time series) to its end (average of the measure over the last tenth of its time series) were considered, **B**=baseline condition. **D**=sleep deprived condition; **Δ=** D-B; * Wilcoxon signed rank test.

Table S4 - Physiological parameters recorded during the Continuous Compensatory Tracker

Physiological parameters[#]	Condition	m ± s.e.	Δ (m ± s.e.)	Sum of positive ranks	p-value*	Effect size
zfT	B	0.02 ± 0.016	0.008 ± 0.18	61.5	.932	-0.01
	D	0.034 ± 0.013				
MaxT	B	0.09 ± 0.01	-0.001 ± 0.014	35.5	.783	-0.05
	D	0.09 ± 0.02				
HR	B	-0.006± 0.01	-0.016 ± 0.017	48	.496	-0.124
	D	-0.023 ± 0.009				
HMA	B	-3.56 ± 2.68	4.83 ± 3.08	82	.211	-0.228
	D	1.27 ± 1.26				

[#] for each measure, the changes from the beginning of the task (average of the measure over the first tenth of its time series) to its end (average of the measure over the last tenth of its time series) were considered, [1] **B**=baseline condition. **D**=sleep deprived condition; **Δ=** D-B; * Wilcoxon signed rank test.
